# The role of the GP in managing suspected transient ischaemic attack: a qualitative study

**DOI:** 10.1186/s12875-019-0963-2

**Published:** 2019-05-21

**Authors:** Duncan Edwards, Grace M. Turner, Satnam K. Virdee, Jonathan Mant

**Affiliations:** 1 Primary Care Unit, Department of Public Health and Primary Care, University of Cambridge, Strangeways Research Laboratory, Worts’ Causeway, Cambridge, CB1 8RN UK; 2 Institute for Applied Health Research, University of Birmingham, Birmingham, Edgbaston B15 2TT UK

**Keywords:** Transient ischaemic attack (TIA), Stroke, General practitioners (GPs), Primary care

## Abstract

**Background:**

National guidelines recommend patients with suspected transient ischaemic attack (TIA) should be seen by a specialist within 24 h. However, people with suspected TIA often present to non-specialised services, particularly primary care. Therefore, general practitioners (GPs) have a crucial role in recognition and urgent referral of people with suspected TIA. This study aims to explore the role of GPs in the initial management of suspected TIA in the United Kingdom (UK).

**Methods:**

One-to-one, semi-structured interviews with GPs, TIA clinic staff and patients with suspected TIA from two sites in the UK: Cambridge and Birmingham. Thematic analysis was undertaken to explore views on the role of the GP in managing suspected TIA. Thirty semi-structured interviews were conducted with stroke patients (*n* = 12), GPs (*n* = 9) and TIA clinic hospital staff (*n* = 9) from two hospitals and nine GP practices in surrounding areas.

**Results:**

Three overarching themes were identified: (1) multiple management pathways for suspected TIA; (2) uncertainty regarding suspected TIA as an emergency or routine situation; and (3) influences on the urgency of GP management.

**Conclusions:**

Guidelines on the primary care management of TIA describe only a small proportion of the factors which influence GP management and referral of suspected TIA. Efforts to improve treatment, appropriate referral and patient experience should use a real rather than idealised model of the GP role in managing suspected TIA.

**Electronic supplementary material:**

The online version of this article (10.1186/s12875-019-0963-2) contains supplementary material, which is available to authorized users.

## Background

Transient ischaemic attack (TIA) occurs when blood flow to the brain is temporarily disrupted; symptoms are similar to a full stroke but short lasting, such a weakness, speech and vison disturbance [1, 2]. TIA is important because patients are at high risk of full stroke, particularly within the first week, which could be fatal or permanently disabling [3]; however, urgent treatment can reduce stroke risk [4].

Evidence-based guidelines recommend that patients with suspected TIA should be treated with aspirin immediately and assessed by a specialist physician in a neurovascular clinic or an acute stroke unit [5]. Previous guidelines stratified patients by risk and recommended those at high risk should be seen by a specialist within 24 h and those at lower risk could be seen within seven days [6], with more recent guidelines (2016) going further and recommending all patient are seen by a specialist within 24 h [5]. However, Amarenco et at (2016) found, even in specialist sites dedicated to urgent evaluation, 21.6% of referrals were not seen by a specialist within 24 h of symptom onset [7].

Public Health media campaigns, such as Act FAST (Face, Arms, Speech, Time), aim to educate the public to recognise stroke symptoms and contact emergency services [8, 9]. However, TIA symptoms are often under-recognised due to their transient nature [10] and because some symptoms, such as non-focal symptoms, are not incorporated in the FAST acronym [2]. People with suspected TIA present to non-specialised services [11, 12] with general practitioners (GPs) being the most frequent first point of contact for medical assistance [13]. Therefore, primary care has a crucial role in the recognition and referral of people with suspected TIA or minor stroke. This study aims to explore the role of GPs in the initial management of suspected TIA in the United Kingdom (UK).

## Methods

### Study design

One-to-one, semi-structured interviews with GPs, TIA clinic staff and patients with suspected TIA from two sites (Birmingham and Cambridge). These were proposed sites for a pilot trial of increased primary care involvement in early treatment of TIA [14]. Qualitative methodology was used as it is best placed to describe participants’ views and experiences of disease, its impact and related healthcare [15].

The objectives were:

1. To establish an understanding of the impact and meaning of a suspected diagnosis of TIA (which included patients who were eventually diagnosed with TIA, minor stroke or neither);
2. To understand the experiences of both patients and medical professionals along the pathway from GP consultation to TIA clinic referral and attendance;
3. To ascertain patients’, GPs’ and TIA clinic staffs’ views on current management of suspected TIA by GPs.

### Participants and setting

Patients were invited to consent to be contacted by the research team by TIA clinic staff at the Queen Elizabeth Hospital (Birmingham) or Addenbrooke’s Hospital (Cambridge) between October 2009 and April 2010. These patients were then purposively sampled according to region, whether they lived alone and final diagnosis. All twelve patients that were telephoned by the research team agreed to be interviewed within three months of clinic attendance. GPs were recruited from nine GP practices that were within the catchment area of the participating TIA clinics, via primary care research networks. Hospital staff interviewees were identified by prior informants using a snowball approach to maximise participation and ensure key informants based in the primary-secondary care interface were identified.

### Data collection and analysis

All interviews were face-to-face, took approximately one hour, and took place in participants’ home (patients) or their place of work (GPs and hospital staff). Field notes were not taken. Interviews were conducted by DE, a GP in Cambridgeshire (*n* = 16); SKV, an experienced qualitative researcher and non-clinician (*n* = 10); NM, a GP in Cambridgeshire (*n* = 3); and SC, an experienced qualitative researcher and non-clinician (*n* = 1). A topic guide was used to structure interviews (see Additional file 1). Data saturation was not formally measured and the sample size was pragmatic on time and resources.

Interviews were audio-recorded and transcribed verbatim. Interviewers checked transcriptions for accuracy but transcripts were not returned to participants. Data collection and analysis ran concurrently with initial analysis guiding further interviews and sampling strategies. Transcripts were thematically analysed by two coders (DE and SE). A subset of the transcripts were coded independently to ensure reliability. NVivo was used to manage the data.

### Ethical considerations

Favourable ethical opinion was given by the Warwickshire Research Ethics Committee (reference 09/H1211/80). Participants were provided with information sheets about the study more than 24 h prior to their interview. Written informed consent was obtained from participants, by the interviewer, immediately prior to the interview.

## Results

Thirty semi-structured interviews were conducted with stroke patients (*n* = 12), GPs (*n* = 9) and TIA clinic hospital staff (*n* = 9) from two hospitals (Cambridge and Birmingham) and nine GP practices in surrounding areas. The median age of the suspected TIA patients was 70 years (interquartile range 53 to 75) and 42% (5/12) were male (Table 1). TIA clinic hospital staff comprised: four stroke nurses, two stroke consultants, two ultrasonographers and one administrator. Five of these participants were from Cambridge and four from Birmingham. The majority of GPs were recruited from Cambridge (6/9) (Table 2).

**Table 1:** Characteristics of patient participants (*n* = 12)

Variable	Stroke patients (*n* = 12)
Age (years)	
Median [Interquartile range]	70 [53, 75]
Range	38–86
Sex	
Male	5
Female	7
Hospital location	
Cambridge	6
Birmingham	6
Referral to TIA clinic	
GP	9
A&E	2
Eye clinic	1
Final diagnosis	
TIA	2
Minor stroke	5
Not stroke/TIA	5
Living status	
Living alone	9
Living with a partner	3

**Table 2:** Characteristics of hospital staff (*n* = 9) and GP participants (*n* = 9)

	Hospital staff	GPs
Sex		
Male	2	6
Female	7	3
Hospital location		
Cambridge	5	6
Birmingham	4	3

Three overarching themes were identified [1]: multiple management pathways for suspected TIA [2]; uncertainty regarding suspected TIA as an emergency or routine situation; and [3] influences on the urgency of GP management.

### Theme 1: multiple management pathways for suspected TIA

The national clinical guidelines at the time of the interviews recommend that people with suspected TIA should be referred for treatment in a specialist clinic within 24 h or seven days depending on ABCD2 score [6]. However, the interviews reported a variety of other management pathways (Table 3); indeed, some GPs could only recall patients taking these “different” pathways:

*“Actually I have referred someone for a TIA. No, he had a TIA and he went through the eye clinic… he came round a different way.”* Cambridge GP 2

In addition, the GPs and hospital staff identified that there were many “inappropriate” or “challenging” patients who were referred to TIA clinics but were unlikely to receive a final diagnosis of TIA or minor stroke. Explanations for these referrals included the GP’s lack of familiarity with TIA, with the patient, or as part of a strategy employed by anxious patients or GPs to get patients seen quicker by a specialist.

*“The other big problem we have is of course the number of mimics that come to clinic and that obviously gets in the way of seeing the genuine TIAs as your clinics are full of mimics, and you can try and educate the GPs but I think that would require a lot of effort, and if they’re only seeing one or two a year then they’re not going to maintain those sort of skills.”* Stroke consultant

*“Because we’re a fast access clinic, they know we’re a quick access to get into the hospital and they think that if we’re not the right people then we’ll refer them on to the right ones.”* Senior TIA specialist nurse

*“The patient I referred is a challenging patient who was getting in essence bad migraines, but she had features that concerned us sufficiently to think it may be TIA – she was getting speech disturbance and facial symptoms as well as visual symptoms and headaches, and I think she saw one of my partners and a referral had been done. She hadn’t got the appointment through, so that’s when I redid the referral and sent it off to the TIA clinic.”* Birmingham GP 1

A number of reasons were identified for why patients were not referred to or did not attend TIA clinics, including other medical conditions such as terminal cancer; patients already being on the maximum preventative treatment they needed/ wanted; transport issues; and patients declining or prioritising other commitments, such as shopping, local festivals and dentist appointments.

*“Some of the elder patients have their shopping day, they’re feeling fine, which they generally are with TIA if they’ve had a little episode and they feel alright afterwards. ‘Oh, I’m not coming in’, they say [when telephoned] even though I stress to them it’s actually quite important that you come in.”* TIA clinic administrator

Most patients, particularly those that lived with a partner, described discussing symptoms with someone else before contacting their GP or accident and emergency (A&E). Patients who lived with partners had additional support preparing for and after a GP appointment, whereas those who lived without partners described substantial worry and practical difficulties in the period between GP assessment and hospital clinic attendance. One patient (Birmingham patient 707X501, age 62, not TIA/stroke, lives alone) described herself feeling “like a lunatic” during these days and described the difficulty of not being able to drive (patients with suspected TIA are banned from driving) to her specialist appointment.

**Table 3:** Referral pathways described by interviewees

Interviewee described referral pathways	
A&E via emergency ambulance	
A&E via taxi	
Hospital TIA clinic within 24 h	
Hospital TIA clinic within 7 days	
Immediate referral by GP, seen by hospital TIA clinic within 2 weeks	
Letter sent by mail days after GP appointment, seen by hospital TIA clinic within 4–6 weeks	
Hospital TIA clinic appointment provided within seven days, rebooked for a later date by patient due to lack of transport or inconvenience	
Neurology, elderly medicine, ENT, vascular medicine or ophthalmology clinic within months	
Referred to hospital TIA clinic but doesn’t attend	
Remained under GP care	
Returned to GP a second or further time for referral	
Referred by out of hours GP to day time GP	

*A&E* Accident and emergency, *ENT* Ear, nose and throat, *GPs* General practitioners, *TIA* Transient ischaemic attack

### Theme 2: uncertainty regarding suspected TIA as an emergency or routine situation

Interviewees from all three groups commented that suspected TIA required urgent action and referral from a GP. Medical staff suggested this was a significant change from the past, when even many full strokes would not receive urgent or intensive management. However, a lack of urgency was reported by some patients, who were “surprised” when the GP wanted to refer them urgently, and some GPs, who suggested that it didn’t always “feel right” to be treating well patients urgently.

*“I always found it a little bit difficult to know how to prioritise TIA in terms of urgency, because it didn’t feel right to send somebody up to hospital very urgently who seems perfectly alright.”* Cambridge GP 3

GPs reported that not all patients seemed to fit guidelines, either because they contacted the GP very soon during the TIA (when a persistent stroke remained a possibility), or long after.

*“The trouble is that sometimes patients will come in a few days after they’ve had an event, they don’t always present the day they have it (laughs), so I think that’s sometimes why it makes it a bit more difficult to sort of refer them urgently. That’s the only thing that it doesn’t say on the proforma, that’s a grey window, because I find difficulties if they’ve had what sounds like symptoms of a TIA a week ago and they say everything is completely resolved within that 24 hour window; I think that’s when patients find it quite difficult if you send them off as an emergency that day.”* Cambridge GP 1

One patient, who had experienced many past TIAs, questioned whether the guidelines were appropriate for her.

*“What does immediately mean? You see, if it happens on a Saturday evening, I mean I wouldn’t go into hospital then would I?”* Birmingham patient (age 84, diagnosed TIA, lives alone)

### Theme 3: influences on the urgency of GP management

Patients, GPs and hospital professionals reported a large variety of factors influencing the GPs role and urgency of GP referral. These factors could be categorised as: patients’ clinical characteristics; general healthcare beliefs; TIA beliefs; patient and GP personalities and relationships; and availability of support tools (Table 4).

**Table 4:** Examples of factors influencing the role of the GP and urgency of GP referral. Examples in bold were listed by patients and GPs. (Parentheses indicate factor was only listed by GPs, patients or hospital staff)

Factors influencing the role of the GP and urgency of GP referral	Examples
Clinical characteristics	• **Established risk factors for TIA severity**^**a**^ • **Symptom un/usual for this patient** • Patient is a frequent attender (GP) • **Additional illnesses** • Frailty or age (GP) • **Family history** • Day of the week and time of day that symptoms occur (patient) • Duration between symptom onset and GP appointment (GP)
General health beliefs and knowledge	• **General health educational level/competence** • **Urgent cases need to go to A&E** • **Urgent cases can wait for up to two weeks** • **A specialist will be best placed to treat patients for a specific disorder** • **Hospital investigations should precede diagnosis and treatment** • Patient choice/demand (GP)
TIA beliefs and knowledge	• **GP and patient knowledge of and belief in national guidelines/awareness campaigns** • GP knowledge of local guidelines and referral pathways (GP) • **If patient feels well it is not an emergency** • **A TIA is like a heart attack** • **Possible brain problems require brain scanning before treatment**
Personalities and relationships	• **Anxious or concerned patient or GP** • **Unconcerned or “not wanting to be a nuisance” patient** • **GP and patient know each other** • GP and patient speak the same language (A&E nurse)
Support tools	• Referral forms (GP) • **Hospital guidelines/ website** • **Telephone and fax access to hospital team** • **GP actions have the “hospital stamp of approval”** • **Availability of investigations such as carotid ultrasound and heart monitors** • **Hospital or other transport** • **Availability of specialist clinics**

^a^Age; blood pressure; weakness or loss of sensation; altered speech; diabetes; previous stroke, TIA or cardiovascular disease; cholesterol; smoking

In terms of clinical features, those featured in guidelines (age, blood pressure, weakness or loss of sensation, altered speech, and diabetes) were listed by all interviewee groups as important in determining the urgency of GP referral, particularly if there were more of them. Patients also listed the following factors: family history; day of the week (weekend symptoms being associated with lower urgency due to a perceived lack of services); their immune system; and if symptom was usual for them. GPs also listed: patient’s other illnesses; frailty; time since the patient’s symptoms occurred; and whether the patient had had similar symptoms before or was a frequent attender.

Perceptions of patients’ general healthcare knowledge (and lack of) was discussed by GPs and vice versa. Patient choice was mentioned by GPs as a reason to manage cases differently from “ideal” management. There was a perception from patients that, as GPs are generalists, specialist care from the hospital is preferable for specific disorders and GPs should defer treatment, such as aspirin, until the patient had been seen by a specialist. GPs reported a similar preference in circumstances such as uncertainty.

*“GPs have a wide range of knowledge, but it’s not necessarily specialised in one particular area, so if treatments were started too soon it might be a detrimental effect.”* Cambridge patient (age 53, not TIA/stroke, lives with partner)

*“If there’s some uncertainty about it and you’re not convinced then, and you can justify it, then you don’t start [medications] until they’ve had additional tests.”* Birmingham GP 1

Interviewees from all three groups cited knowledge of TIA as important to ensure acceptable GP management of suspected TIA, though patients tended to discuss this in the context of being a “good” GP in general, and some GPs were dismissive of whether knowledge of guidelines was an important or realistic way to inform their practice. Lack of urgency was influenced by GPs’ view that patients are well enough if they come into a general practice, and patients’ perception of transient symptoms. In contrast, concerns that the symptoms could be due to a bleed on the brain and considering TIA analogous to heart attack were related to a perception that urgent hospitalisation could be required.

*“I guess by definition most patients who come in to general practice are well by hospital terms, they’ve managed to get up to the practice, walk in.”* Birmingham GP 1

*“The numbness went off reasonably quickly and I thought, "well, that's it, that's the end of it" sort of thing.”* Cambridge patient (age 86, TIA, lives with partner)

The personalities of GPs and patients was discussed as influencing GPs decision making. Patient or GP anxiety caused more urgent action; however, one patient described wanting not to be a “nuisance”. Balancing communicating the seriousness of suspected TIA with avoiding raising too much anxiety was described as “endlessly difficult” by one GP. Many patients felt GPs were better able to manage any health problem including suspected TIA when they knew the GP; however, some patients and most GPs felt continuity of care made little difference and some patients prioritised specialist knowledge over continuity of care.

GPs and hospital staff suggested support tools such as hospital websites, hospital referral proformas and guidelines altered the GP’s management and the urgency or referral. Environmental factors, such as availability of a fax machines or telephones, influenced communication between the GP and hospital, and subsequent speed of referral. This also enhanced patients’ satisfaction as the information exchange with secondary care was perceived as a hospital stamp of approval. However, transport to hospital clinics at short notice was a barrier identified by all three groups and some GPs highlighted the lack of availability of weekend clinics.

## Discussion

Our findings identified multiple different management pathways for suspected TIA other than the guideline recommendations; 12 different referral pathways were described. There is uncertainty among patients and GPs regarding whether suspected TIA should be treated as an emergency, routine, or somewhere in between. Some participants (both patients and healthcare professionals) recognised the need for urgent action; however, others were hesitant in their actions due to the transient nature of symptoms and a wide variety of additional factors. The many factors which influenced the urgency of GP referral included: patients’ clinical characteristics, general healthcare beliefs, TIA beliefs, patients and GPs personalities and relationships, and availability of support tools.

Mellor et al. (for stroke) [16] and Wilson et al. (for TIA and minor stroke) [17] have also described how multiple pathways occur for patients accessing specialist stroke services and our study supports their findings. Variations in patients and GPs perception of urgency have been described by Mc Sharry et al. (2014) who found that perceived lack of urgency from first point of contact healthcare providers resulted in delayed help seeking behaviour from patients [18]. We identified support tools as facilitators for healthcare providers’ decision-making for referrals to specialist services. Other qualitative work has described variations in use of support tools by GPs, such as the ABCD2 score, with some GPs disregarding scores if they conflicted with their clinical judgment/ experience [17].

Despite media campaigns aimed at educating the public to recognise symptoms of stroke and to contact emergency services, the ability of the general public to recognise and respond urgently to stroke/TIA symptoms remains limited [19]. A systematic review found recognition of symptoms did not reduce time to presentation and GPs were frequently the first point of contact [13]. Therefore, the role of GPs in the initial management of suspected TIA remains relevant and important. Other studies have reported service factor barriers to urgent referral of suspected TIA from primary care to specialist services, including difficulties in getting urgent primary care appointments and lack of recognition of symptoms from GP practice receptionists [17]. However, our study has identified there are many different factors which influenced the urgency of GP referral: these encompass knowledge and attitudes (TIA specific and general health); GP/ patient relationships; patient factors (clinical characteristics and personalities); and environmental factors (support tools and referral processes). The majority of these factors are not listed in guidelines on the assessment of suspected TIA, which focus on neurological symptoms and cardiovascular risk factors [5, 20]. These results highlight the complexity of improving the management of people with suspected TIA who present in primary care. Future interventions require a multifaceted approach which include consideration of context-specific and individual factors.

A key strength of this study is that, opposed to other studies which focus on referral pathways for people with a definite TIA diagnosis, the sampling within our study enabled exploration of referral decisions for patients who were eventually diagnosed with other conditions and (via GP interviews) patients who were never referred to secondary care. Furthermore, similar studies have focused on the patients’ perspective [16, 18]; however, our study also included perspectives of GPs and TIA clinic staff. Participants were recruited from two sites (Birmingham and Cambridge) and there were similarities between themes at each site suggesting the findings may be relevant to other areas in the UK.

A limitation is that data saturation was not formally measured [21] and the sample size was pragmatic based on time and resources. However, the study aimed to sample a broad range of participants and sufficient elucidation of major themes was prioritised. Sampling across three groups (patients, GPs and hospital professionals) enabled inter-group comparisons to be made. We were unable to collect information on characteristics of patients who declined to participate in the study as all these patients declined to provide details of their case. Therefore, it is difficult to determine if non-participants were different from those that participated. The interviews were conducted by both GPs (DE and NM, *n* = 19 interviews) and non-clinicians (SKV and SC, *n* = 11 interviews). There are advantages and disadvantages of interviews being conducted by clinicians and non-clinicians. Professional similarity can result in interviews that are “broader in scope and provided richer and more personal accounts of attitudes and behaviour in clinical practice” [22]. However, professional similarity also raises the possibility of shared attitudes and biases between interviewer and interviewees. Discussion amongst the four interviewers was used to limit this bias. It is important to note that at the time of the interviews the guidelines stratified patients by risk and recommended those at high risk should be seen by a specialist within 24 h and those at lower risk could be seen within seven days [6]. These guidelines were revised in 2016 to recommend all patient are seen by a specialist urgently, within 24 h [5].

## Conclusion

Guidelines on the primary care management of TIA describe only a small proportion of factors which influence GP management and referral of suspected TIA. This study demonstrates a much wider variety of factors that significantly impact primary care management of suspected TIA, and demonstrates the diversity of referral pathways that occur in real-life clinical practice. Efforts to improve treatment, appropriate referral and patient experience should use a real rather than idealised model of the GP role in managing suspected TIA.

## Additional file

Additional file 1: Semi-structured interview prompts for RAPID-TIA Qualitative Study. (DOCX 21 kb)

## Abbreviations

A&E: Accident and emergency
ENT: Ear, nose and throat
FAST: Face, arms, speech, time
GPs: General practitioners
TIA: Transient ischaemic attack
UK: United Kingdom
