# Supplementary material for: The role of the GP in managing suspected transient ischaemic attack: a qualitative study
Source: BMC Fam Pract. 2019 May 21;20:67. doi: 10.1186/s12875-019-0963-2 (PMC6530060; doi:10.1186/s12875-019-0963-2)
Supplement: Supplementary file 1 — Semi-structured interview prompts for RAPID-TIA Qualitative Study. (DOCX 21 kb) [file 12875_2019_963_MOESM1_ESM.docx]

***Additional file 1***

***Semi-structured interview prompts for RAPID-TIA Qualitative Study***

**Patient interview prompts**

*Post TIA clinic, general retrospective interview.*

1. Why do you think you are here?

a. So, what exactly has happened to you?

2. How are you feeling at the moment?

a. Are you well?

b. Are you ill?

c. How would you describe your state of health right now?

3. Can you go back and describe what happened to you, before going to see the GP?

a. What did you think might be wrong with you?

b. Did you talk to people about it?

c. Did you look up information anywhere?

4. Did you see your regular GP? Did that make a difference?

5. What did the GP tell you?

a. What did he/she say had happened?

b. Did the GP give it a name?

c. – if named - Had you ever heard of this (eg TIA, or the phrase ‘mini-stroke’) before?

d. What other things were you told?

6. How did you feel after seeing the GP?

a. Did you talk to relatives or friends?

b. Did the GP give you any medication?

c. Did the GP suggest or instruct you should do anything else?

d. Have you changed your behaviour yourself in anyway?

7. Did you have to wait for an appointment to come to the clinic?

a. How long?

b. Does that seem about right to you?

c. How did you feel between seeing the GP and coming to the clinic?

8. What was it like coming to the clinic? What were the main differences from when you saw the GP?

9. Did the GP and the clinic handover all the important details about you throughout?

10. What new things did you learn at the clinic?

a. Did you have any tests done? What tests were they?

i. How do you feel about them?

b. Have you been given any new medication?

c. How do you feel about having these tests/medications?

11. How do you feel now that you have seen someone at the hospital?

a. Are you worried or anxious about anything at this stage?

b. What, if anything, happens now?

12. We’re interested in the differences between GP and hospital care for treating people like you.

a. Do you think there should be any change in the way things work between the GP and the hospital?

b. What do you think about the idea of changing things – perhaps giving the GP more of a role so that things might happen just a little earlier?

c. What if the GP started you on medication before you came to the hospital?

d. What about not having tests yet?

13. Do you have a clear sense of what happened to you? Do you think the GP and the clinic understand what happened to you in the same way, or are there any differences?

14. If there’s a possibility of improving treatment of TIA, we would have to test it first. This means some people would be given the new treatment and some people would be given the current treatment, but neither the patient nor his GP would be able to choose.

a. People who choose to participate in the trial would be randomly selected to be given the new treatment or the current treatment – what’s your feeling about this?

b. Whichever treatment is given, it would need to be given immediately – how much information should be given to patients?

c. Because quick treatment is crucial, it would be the GP who was helping a person decide right there and then whether to take part in the research – what’s your feeling about this?

d. Being part of this research would mean half the people get the new treatment and half the people get the current treatment – is that okay?

**GP interview prompts**

1. Why do you refer patients to the TIA clinic?

a. What things do you do before making a referral to the TIA clinic?

b. What criteria do you personally draw on in order to decide whether to refer or not?

c. Do you routinely use the ABCD2 score? How is it helpful?

d. How much do you say to patients and how much do you leave for the clinic?

2. How do you interact with patients who you suspect have had a TIA?

a. Are they well?

b. Are they ill?

3. Specifically for patients with suspected TIA, is continuity of care a significant issue?

a. i.e. is it important they see their “own” GP?

b. i.e. is it important they are seen at their regular practice rather than out of hours?

c. i.e. is it important they are seen at their regular practice rather than in A&E?

4. Do you feel patients have to wait long to be seen at the TIA clinic?

a. Does it make sense for patients to have different waiting times?

b. Are you aware of difficulties some patients have with waiting?

5. What do you think your role is when seeing patients with suspected TIA?

6. Could you tell me what your relationship with TIA clinic is like?

a. Are there particular people at the clinic that you have a working relationship with?

b. Where else might you send patients that you don’t send to the TIA clinic?

7. Do you know what happens to patients when you refer them to the TIA clinic?

a. How do you normally hear back from the clinic?

8. What happens after the patient is discharged from the clinic?

9. Do you currently ask some patients with suspected TIA to take any new medications or to do anything differently?

10. We’re interested in the differences between GP and hospital care for treating patients with suspected TIA.

a. Do you think there should be any change in the way things work between the GP and the hospital?

b. What do you think about the idea of changing things – perhaps having more of a role so that things might happen a little earlier?

c. What if you started patients on extra medications before they came to the hospital e.g. adding dipyradamole, a statin and aggressive BP control before they go to the hospital?

d. Can you see any problems or advantages with this?

11. How comfortable are you with deciding a patient needs extra medications for a suspected TIA?

a. How do you feel about starting these before they’ve had any tests?

b. How comfortable are you with the TIA clinic starting a patient on life long medications based on their diagnosis of TIA?

12. A future trial will be looking at improving treatment of TIA through getting GPs to prescribe extra medications for half the patients in the study.

a. Patients will be randomised to be given the new treatment or the current treatment – what’s your feeling about this?

b. Whichever treatment is given, it would need to be given immediately – how much information should be given to patients?

c. Because quick treatment is crucial, it would be the GP who was helping the patient decide right there and then whether to take part in the research – what’s your feeling about this?

d. Would you experience any dilemma in randomising the treatment of your patient?

e. The trial would require the GP to fill in a form, much like the current referral proforma, phone a telephone number (for the randomization), invite the patient to participate, and treat the patient. What is the best way of doing this?

**Hospital staff interview prompts**

1. Why are patients referred to the TIA clinic?

a. By whom are patients referred?

b. What are the reasons patients themselves attend the TIA clinic?

c. What things do referrers do before making a referral to the TIA clinic?

d. Do referrers routinely use the ABCD2 score? How is it helpful?

e. How much do GPs say to patients and how much do they leave for the clinic?

2. How do you interact with patients who you suspect have had a TIA?

a. Are they well?

b. Are they ill?

3. Specifically for patients with suspected TIA, is continuity of care a significant issue?

a. i.e. is it important they see their “own” GP?

b. i.e. is it different if they are seen at their regular GP practice rather than in A&E?

4. Do you feel patients have to wait long to be seen at the TIA clinic?

a. Does it make sense for patients to have different waiting times?

b. Are you aware of difficulties some patients have with waiting?

5. What do you think your role is when seeing patients with suspected TIA?

6. Do you work well together with GPs? How about A&E and other referrers?

7. What happens after the patient is discharged from the clinic?

8. Do GPs currently ask some patients with suspected TIA to take any new medications or to do anything differently?

9. We’re interested in the differences between GP and hospital care for treating patients with suspected TIA.

a. Do you think there should be any change in the way things work between the GP and the hospital?

b. What do you think about the idea of changing things – perhaps giving GPs more of a role so that things might happen a little earlier?

c. What if GPs started patients on extra medications before they came to the hospital e.g. adding dipyradamole, a statin and aggressive BP control before they go to the hospital?

d. Can you see any problems or advantages with this?

10. How happy or comfortable are you with GPs deciding a patient needs extra medications for a suspected TIA?

a. How do you feel about GPs starting these before they’ve had any tests?

b. How comfortable are you yourself in starting a patient on life long medications for a TIA?

11. A future study will be looking at improving treatment of TIA through getting GPs to prescribe extra medications for half the patients in the study.

a. Patients will be randomised to be given the new treatment or the current treatment – what’s your feeling about this?

b. Whichever treatment is given, it would need to be given immediately by the GP – how much information should be given to patients?

c. Because quick treatment is crucial, it would be the GP who was helping the patient decide right there and then whether to take part in the research – what’s your feeling about this?
